# Factors Influencing Hemoglobin Variability and Its Association with Mortality in Hemodialysis Patients

**DOI:** 10.1155/2018/8065691

**Published:** 2018-04-01

**Authors:** Zeynep Bal, Bahar Gurlek Demirci, Suleyman Karakose, Emre Tutal, Mehtap Erkmen Uyar, Nurhan Ozdemir Acar, Siren Sezer

**Affiliations:** ^1^Department of Nephrology, Ankara Training and Research Hospital, Ankara, Turkey; ^2^Dr. Nafiz Korez State Hospital, Ankara, Turkey; ^3^Department of Nephrology, Baskent University Faculty of Medicine, Ankara, Turkey; ^4^Department of Nephrology, Baskent University Faculty of Medicine, Istanbul, Turkey

## Abstract

**Purpose:**

We aimed to investigate the factors influencing hemoglobin variability with inflammatory and nutritional parameters and its associations with all-cause mortality among hemodialysis patients.

**Methods:**

One hundred and sixty-nine patients during the entire 12 months were enrolled into the study. Fasting plasma glucose, creatinine, calcium, phosphorus, alkaline phosphatase, parathyroid hormone (PTH), C-reactive protein (CRP), serum iron, serum iron-binding capacity, and transferrin saturation were analyzed. We defined six groups: low, target range, high, low-amplitude fluctuation with low hemoglobin levels, low-amplitude fluctuation with high hemoglobin levels, and high-amplitude fluctuation. Body mass index (BMI), malnutrition-inflammation score (MIS), and Charlson Comorbidity Index were evaluated.

**Results:**

Hemoglobin variability was significantly correlated with age, platelet count, and number of hospitalization instances and inversely correlated with erythropoietin dose per body surface area. The coefficient of variation of hemoglobin showed a correlation with MIS and ferritin. The absolute level of hemoglobin showed a negative correlation between PTH, CRP, MIS, number of hospitalization instances and a positive correlation with albumin and BMI. High, low, and target-range groups showed survival advantage compared to the other three groups. In regression analysis, age, CRP levels, MIS, and BMI were the predictors of mortality.

**Conclusion:**

Inflammation and duration of anemia were the major predictors of hemoglobin variability. High-amplitude fluctuation predicts high mortality; on the contrary low-amplitude fluctuations is related to better survival. MIS was independently associated with mortality. This trial is registered with NCT03454906.

## 1. Introduction

Anemia is a common complication of chronic kidney disease (CKD). The introduction of erythropoiesis-stimulating agents (ESA) has led to a dramatic reduction in blood transfusion requirements and has been associated with improved quality of life [1, 2]. Although the optimal target hemoglobin concentration in CKD remains a matter of considerable uncertainty, the European Best Practice Guidelines (EBPG) recommended that the target hemoglobin level should be defined on an individual basis, taking into account gender, age, ethnicity, activity, and comorbid conditions [3].

Fluctuation in hemoglobin levels, known as hemoglobin variability during treatment with ESA, is a well-documented phenomenon [4]. Several factors have been found to affect hemoglobin variability, including drug-related issues, such as pharmacokinetic parameters, patient-related differences in demographic characteristics, and factors affecting clinical status, as well as clinical practice guidelines, treatment protocols, and reimbursement policies [5]. Inflammation is also an important factor associated with hemoglobin variability, and the consequences of persistent inflammatory activity are far-reaching in affected patients. Lacson Jr. et al. [6] reported that few patients maintained hemoglobin in the target range in a study that measured variability in patients with CKD. Overall, only 38.4% of patients had hemoglobin levels between 11 and 12 g/dL, while 12.2%, 31.5%, and 30% of patients had hemoglobin levels at 10, <11, and >12 g/dL, respectively. Ebben et al. measured hemoglobin levels in a large cohort of patients for 6 months and found that only a minority of patients (6.5%) had stable hemoglobin levels over the study period with nearly 90% exhibiting some pattern of hemoglobin fluctuation [7].

In the present study, we aimed to investigate factors influencing hemoglobin variability with inflammatory and nutritional parameters and the association of hemoglobin variability with all-cause mortality among hemodialysis patients.

## 2. Materials and Methods

This was a single-center study at a university teaching hospital. Among 220 hemodialysis patients, 169 patients (107 males; aged 54.3 ± 14.5 years) during the entire 12 months (January 2009 to January 2010) were enrolled into the study. The dosage of ESA was assessed due to reimbursement from our country as 150 U/kg/week if hemoglobin level was <11 mg/dl and 75 U/kg/week if hemoglobin level was between 11 and 11.9 mg/dl. ESA therapy was stopped when hemoglobin level reached 12 mg/dl. Intravenous iron sucrose was prescribed if ferritin was <100 *μ*g/L or the transferrin saturation (TSAT) was <20% and Hgb was below the target range. Patients received 100 mg intravenously over each of the next 10 HD treatments and then every 2 weeks thereafter. Iron was withheld if ferritin was >800 *μ*g/L or the TSAT was >50%.

All patients' standard clinical (age, gender, duration of hemodialysis, and history of hospitalization) and biochemical parameters were analyzed cross-sectionally. As part of our quality assurance program, complete blood count and biochemical parameters were checked during the monthly clinic visit within this period. In all participants, a venous blood sample was collected after an overnight fast to measure the concentration of the following biochemical variables using standard laboratory techniques: fasting plasma glucose (FPG), creatinine, calcium, phosphorus, albumin, alkaline phosphatase (ALP), parathyroid hormone (PTH) levels, C-reactive protein (CRP), iron parameters (serum iron, serum iron-binding capacity, and transferring saturation), and a lipid profile (total cholesterol [C], HDL-C, TGs, and LDL-C [low-density lipoprotein C; computed from Friedewald's formula]). All patients had a minimum of six hemoglobin measurements within a 6-month period. They were excluded if they either received a kidney transplant during this time or had a blood transfusion during the baseline period. Patients with serum ferritin < 100 *μ*g/L were also excluded from the study because of reimbursement from our country. We defined six groups of patients based on their overall pattern of fluctuation to assess both the frequency and size of fluctuations in hemoglobin levels over time: consistently low (L; all 6 months with low hemoglobin levels), consistently within the target range (T; all 6 months with target-range hemoglobin levels), consistently high (H; all 6 months with high hemoglobin levels), low-amplitude fluctuation with low hemoglobin levels (LAL; all 6 months with low or target-range hemoglobin levels), low-amplitude fluctuation with high hemoglobin levels (LAH; all 6 months with target-range or high hemoglobin levels), and high-amplitude fluctuation (HA; low, target-range, and high hemoglobin levels within the 6-month period). ESA and iron therapy dosages were calculated per month and cumulative dose per year.

The number of months that elapsed with hemoglobin < 11 g/dl was also calculated. Body weight and body mass index (BMI; in kg/m^2^) were taken after the dialysis session. Height was obtained from the patient's chart. The malnutrition-inflammation score (MIS) assessment was performed according to the description by Kalantar-Zadeh et al. [8]. It consisted of medical history (weight changes, dietary intake, gastrointestinal symptoms, functional capacity, and presence of comorbidities), physical examination (loss of subcutaneous fat and muscle wasting), and laboratory parameters (serum albumin and total iron-binding capacity). Each of the components was scored from 0 (normal) to 3 (severely abnormal). The sum of all points gave the total MIS (maximum 30).

The Charlson Comorbidity Index (CCI) is based on ICD-9 codes in claims data. It was designed as a measure of the risk of 1- to 10-year mortality attributable to comorbidity in a longitudinal study of general hospitalized patients.

This study was approved by Baskent University Institutional Review Board and Ethics Committee (IRB approval number: KA09/358).

### 2.1. Statistical Analyses

Statistical analyses were performed by using SPSS software (Statistical Package for the Social Sciences, version 11.0, SPSS Inc., Chicago, IL, USA). Normality of data was analyzed by using a Kolmogorov-Smirnov test. All numerical variables with normal distribution were expressed as means ± standard deviations (SD), while variables with skewed distributions were expressed as medians and interquartile ranges (IR). Categorical variables were expressed as percentages and compared by a chi-square test. Normally distributed numeric variables were analyzed by independent samples *t*-tests or one-way ANOVA (post hoc Tukey) tests according to distribution normality. The baseline variables used for analysis were similar to those for survival analysis. A Kaplan–Meier analysis was used to examine the effects of hemoglobin variability on all-cause mortality and the time to ESRD. Descriptive statistics were used to examine patient characteristics according to the six hemoglobin variability categories of the first classification system, and mortality rates were calculated for the categories. A value of *p* < 0.05 was considered statistically significant. Descriptive statistics were used to examine patient characteristics according to the six hemoglobin variability categories of the first classification system, and mortality rates were calculated for the categories.

## 3. Results

Demographic characteristics and mean values for laboratory parameters of the study population during the 12-month period are summarized in Table 1. Using the classification system, the percentage of patients in each of the six groups was as follows: consistently low 21.8%, consistently within the target range 24.8%, consistently high 7.6%, low-amplitude fluctuation with low hemoglobin levels 13.0%, low-amplitude fluctuation with high hemoglobin levels 9.4%, and high-amplitude fluctuation 23.4%. Among these six groups, there was no significant difference with respect to age, duration of hemodialysis, urea reduction ratio, serum PTH, calcium, phosphorus, albumin, iron, and transferrin saturation levels.

The percentage of patients with mean hemoglobin concentration < 11 g/dl was 60.4%. Mean hemoglobin levels differed significantly by gender (10.9 ± 1.2 g/dl [men] versus 10.4 ± 0.96 g/dl [women], *p* = 0.006). The minimum and maximum dosages of ESA were 25 U/kg/week and 150 U/kg/week, respectively. The median dosages of iron therapy per body weight were 15.7 (25), 14.7 (27), 13.4 (16), 16.5 (19), 18.2 (19), and 11.7 (20) mg/dl; BMI levels were 23.3 ± 5.5, 22.7 ± 3.7, 22.4 ± 3.1, 21.7 ± 4.8, 23.3 ± 4.3, and 22.4 ± 4.5 kg/m^2^; MIS levels were 7 (4), 5 (5), 7 (4), 6 (3.5), 7 (2.8), and 7.5 (4) in patient groups with high-amplitude fluctuation, high, target, low, and low-amplitude fluctuation with high hemoglobin level, and low-amplitude fluctuation with low hemoglobin level, respectively (Table 2).

The absolute level of hemoglobin showed a negative correlation between PTH, CRP, MIS, and number of hospitalizations (*p*: 0.028, *p*: 0.004, *p*: 0.009, and *p*: 0.008, resp.) and a positive correlation with albumin and BMI (*p*: 0.007 and *p*: 0.009, resp.). Hemoglobin variability was significantly correlated with age (*p*: 0.007), platelet count (*p*: <0.001), and the number of hospitalizations (*p*: 0.006) and inversely correlated with erythropoietin dose per body surface area (*p*: 0.001). The coefficient of variation of hemoglobin showed a positive correlation with MIS and ferritin (*p*: 0.046 and *p*: 0.026). The duration of dialysis, low levels of leucocyte, ferritin, and CRP, and high doses of erythropoietin were noted as effecting parameters for hemoglobin > 11 g/dl (*p*: 0.002, *p*: 0.005, *p*: <0.001, *p*: 0.042, and *p*: 0.001, resp.).

**Figure 1:**
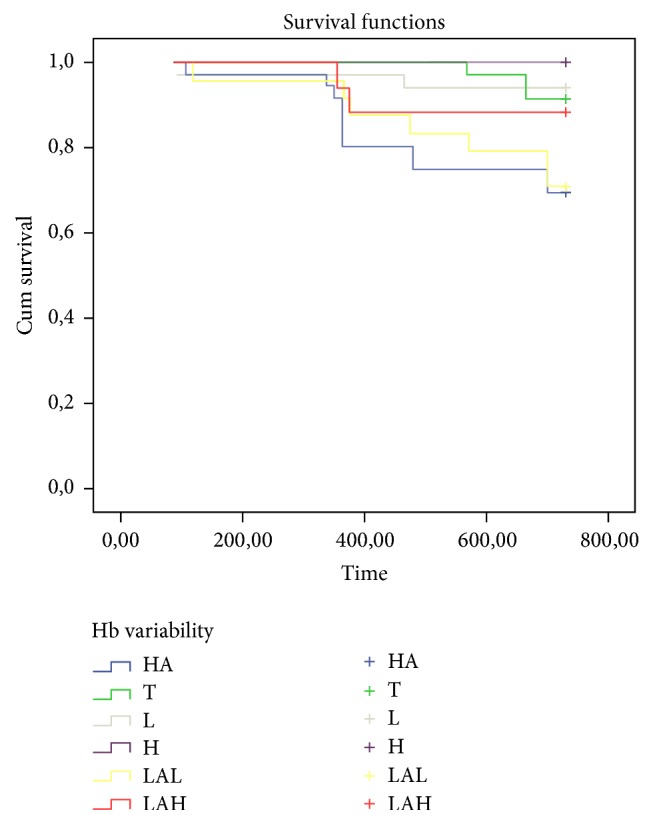
Kaplan–Meier analysis showing significant difference associated with mortality in each of the hemoglobin variability groups. Consistently high (H), consistently low (L), consistently within the target range (T) groups showed survival advantage compared with low-amplitude fluctuation with high hemoglobin levels (LAH), low-amplitude fluctuation with low hemoglobin levels (LAL), and high-amplitude fluctuation (HA) groups (100%, 94.1%, 91.4%, 88%, 70.8%, and 69.4%, resp.; *p* < 0.009).

The number of months elapsed with hemoglobin < 11 g/dl was inversely correlated with the duration of hemodialysis, hematocrit, albumin, and body weight (*p*: 0.002,* p*: <0.001, *p*: 0.003, and *p*: 0.040, resp.) and significantly correlated with ferritin, PTH, total erythropoietin dose, MIS, and number of hospitalizations (*p*: <0.001, *p*: 0.38,* p*: <0.001, *p*: 0.013, and *p*: 0.007, resp.). In addition, the number of months elapsed with hemoglobin < 11 g/dl was significantly different in these six groups as the lowest in the consistently high group (0.5 ± 0.6 months) and the highest in low-amplitude fluctuation with high hemoglobin levels (10.4 ± 1.2 months) (*p* = 0.001). Kaplan–Meier analysis showed a significant difference associated with mortality in each of the hemoglobin variability groups. High, low, and target-range groups showed a survival advantage compared with the low-amplitude fluctuation with high hemoglobin levels, low-amplitude fluctuation with low hemoglobin levels, and high-amplitude fluctuation groups (100%, 94.1%, 91.4%, 88%, 70.8%, and 69.4%, resp.; *p* < 0.009) (Figure 1). In regression analysis, age, CRP levels, MIS, and BMI were significantly important predictors of mortality (*p*: 0.027, *p*: 0.008, *p*: 0.002, and *p*: 0.046, resp.) (Table 3).

## 4. Discussion

In the present study, we detected hemoglobin variability that was significantly lower in patients in the high stable group than in other groups. This difference may be a result of more enhanced renal function in this group even though we did not detect preserved renal function in patients because of limitations of this study. Also, we found that hemoglobin variability has a modest association with morbidity and all-cause mortality in erythropoietin-treated dialysis patients. Although the mechanism of hemoglobin variability remains uncertain, patients treated with ESA pharmacologic features and dosing of ESA are believed to lead to a cyclical pattern of hemoglobin levels within the recommended range [9]. A study showed that only about 5% of CKD patients maintained target levels of hemoglobin after a few months of observation [10]. Another study reported that over 90% of patients experienced hemoglobin cycling [11]. In the literature, studies concerning hemoglobin variability and its association with mortality administered an unlimited ESA dosage to patients. Conversely, in our study, we used stable doses and limited ESA therapy because of medical policy.

The present study also evaluated the relationship between ESA dosage per body weight in specific variability groups. The results were not conflicting with previous studies [12] as ESA doses were highest and increased over time in patients with consistently low hemoglobin levels. Also, doses were lower and decreased in patients with consistently high hemoglobin levels. ESA dose requirements were the lowest for patients with hemoglobin levels consistently within the high stable group. When compared with previous studies, we used relatively lower doses of ESA, and we believe that hemoglobin variability patterns were not related to ESA dosage but might be a result of both intermittent inflammation and dietary factors.

The CHOIR [13] and TREAT [14] trials also showed that not ESA itself but its response rates are predictive for mortality risk factors. In our study, patients in the high stable group had the lowest mortality rate, whereas patients in the high-amplitude fluctuation and low stable groups had the highest mortality rates although hemoglobin variability values did not show significance between deceased or alive patients. This result was not surprising because it has been recognized that more than 50% of patients with CKD have an activated inflammatory response, as shown by increased levels of CRP and other proinflammatory cytokines [15].

Evidence suggests that inflammation is an important factor associated with hemoglobin variability and the CRP level is a good predictor for patients with less stable hemoglobin levels in CKD patients [4]. The studies by Dellanna et al. and Barany et al. both found a significant correlation between hemoglobin variation and CRP values [16, 17]. In our study, patients in the high stable group had lower serum leucocyte and CRP levels that supported both lower inflammatory processes and lower inflammation-related mortality rates. These results provide supporting evidence that inflammation can trigger hemoglobin variability. Thus, ESA dosage should be revised, and patients should be followed closely in the presence of inflammatory conditions. Moreover, inflammation is closely related to protein-energy malnutrition in dialysis patients and the combination of these two conditions, known as the malnutrition-inflammation complex syndrome, is frequently observed in dialysis patients [18]. The inflammatory and nutritional status can be evaluated by the malnutrition-inflammation score (MIS). Our results support previous studies demonstrating that serum CRP and MIS were predictive for both morbidity and mortality [19]. In addition, because we used limited ESA therapy at a maximum of 150 U/kg/week, we did not observe any mortality associated with complications of hemoglobin overshooting, including hypertension, high platelet counts, and thrombotic events [20].

In Eckardt et al.'s study, among 5,037 hemodialysis patients, low levels of hemoglobin (<11 g/dl) were established as an increased risk for death and is similar to our results. Furthermore, a lower body mass index and the timing of hemoglobin values < 11 g/dl were both detected as predictors of mortality of hemoglobin variability supporting our study [21]. Another study by Gilbertson et al. with 159,720 HD patients receiving rHuEPO found that patients who had a low hemoglobin level (<11 g/dl) for the longest time had the highest mortality risk [22]. These findings were also similar to the results of our study. Our study has some limitations. First, because of the impact of medical policy, ESA use was limited. Second, our follow-up period may not have been long enough. Third, our data were collected retrospectively from one medical center and obtained from a single database and, therefore, reflect only a small sample.

In conclusion, our study demonstrates that both inflammation and the duration of anemia were the major predictors of hemoglobin variability. We also disproved that high-amplitude fluctuations predict high mortality, but rather that low-amplitude fluctuations are concerned with better survival rates. In addition, hemoglobin variability itself did not predict mortality; MIS and its effects on hemoglobin variability were independently associated with mortality. Therefore, a large randomized trial is needed to evaluate clinical outcomes of hemoglobin variability in this population.

## Figures and Tables

**Table 1:** Patient characteristics of hemoglobin variability.

Patient characteristics	HA (*n*: 39)	High (*n*: 13)	Target (*n*: 42)	Low (*n*: 37)	LAH (*n*: 16)	LAL (*n*: 22)
% of total (*n*: 169)	23.4	7.6	24.8	21.8	9.4	13
Age (years) (mean ± SD)	54.6 ± 14.0	47.4 ± 8.3	57.8 ± 13.3	51.7 ± 18.3	53.8 ± 12.2	55.2 ± 15.1
Gender (male) (%)	25 (44.7%)	7 (7.6%)	31 (18.3%)	23 (43.6%)	16 (4.7%)	13 (7.6%)
CKD etiology						
Diabetes mellitus	3	2	3	4	2	3
Hypertension	6	3	9	7	3	4
PCKD	3	1	3	4	2	3
Unknown etiology	10	2	9	7	3	5
Others	17	5	18	15	6	7
BMI (kg/m^2^)	23.3 ± 5.5	22.7 ± 3.7	22.4 ± 3.1	21.7 ± 4.8	23.3 ± 4.3	22.4 ± 4.5
Duration of dialysis (years) (mean ± SD)	10.0 ± 5.9	10.2 ± 4.9	9.0 ± 5.7	9.2 ± 5.2	10.0 ± 7.1	5.8 ± 4.7

HA: high-amplitude fluctuation; LAH: low-amplitude fluctuation with high hemoglobin; LAL: low-amplitude fluctuation with low hemoglobin; BMI: body mass index; CKD: chronic kidney disease; PCKD: polycystic kidney disease; SD: standard deviation.

**Table 2:** Biochemical parameters and medications of hemoglobin variability.

Patient characteristics	HA (*n*: 39)	High (*n*: 13)	Target (*n*: 42)	Low (*n*: 37)	LAH (*n*: 16)	LAL (*n*: 22)	*p*
Hemoglobin (g/dl)	10.7 ± 0.7	12.7 ± 0.8	11.1 ± 0.4	9.9 ± 0.8	12.2 ± 1.0	9.6 ± 0.6	**0.001**
Hb-var (g/dl)^*∗*^	1 (0.4)	0.7 (0.3)	0.8 (0.3)	0.8 (0.2)	1 (0.1)	0.9 (0.2)	**0.001**
Maximum Hb-var (g/dl)^*∗*^	4 (1.5)	2.4 (1.2)	2.5 (0.8)	2.6 (1.3)	3.9 (1.1)	3.6 (0.8)	**0.001**
Coefficient of Hb-var^*∗*^	9.3 (4.1)	5.9 (2.8)	7.3 (2.7)	7.8 (2.8)	8.4 (1.5)	10.1 (2.9)	**0.001**
MCV (fl)	90.1 ± 3.9	89.1 ± 4.2	91.2 ± 4.6	91.5 ± 5.2	86.8 ± 6.8	88.1 ± 7.0	**0.017**
RDW (%)^*∗*^	16.5 (2.4)	16.3 (2.2)	15.8 (1.5)	16.0 (1.2)	16.9 (1.6)	16.1 (1.6)	0.085
Leukocyte (×10^3^/*µ*l)	7.0 ± 1.7	6.4 ± 1.3	6.7 ± 1.6	5.9 ± 1.7	7.3 ± 2.1	6.9 ± 1.6	**0.05**
Calcium (mg/dl)	9.4 ± 0.6	9.0 ± 0.6	9.2 ± 0.6	9.2 ± 0.8	9.2 ± 0.9	8.8 ± 0.6	0.135
Phosphate (mg/dl)	5.7 ± 1.5	5.3 ± 0.4	5.0 ± 1.2	5.1 ± 1.4	5.0 ± 1.1	5.1 ± 1.0	0.239
Albumin (g/dl)	4.0 ± 0.3	4.3 ± 0.3	4.1 ± 0.3	4.1 ± 0.3	4.2 ± 0.6	4.1 ± 0.4	0.144
CRP (mg/l)^*∗*^	9.7 (13)	4.7 (6)	9.7 (15)	7.6 (14)	11.2 (10)	7.5 (22)	0.744
Transferrin saturation%^*∗*^	29.7 (12)	35.5 (14)	32.3 (13)	29.2 (23)	28.5 (17)	29.0 (17)	0.907
Ferritin (*µ*g/l)^*∗*^	275 (400)	124 (86)	310 (222)	382 (298)	196 (248)	462 (400)	**0.001**
PTH (pg/ml)^*∗*^	445.7 (728)	303.7 (400)	281.3 (508)	491.2 (1078)	214.4 (515)	299.7 (401)	0.118
URR%	66.2 ± 5.3	65.4 ± 4.6	66.1 ± 6.5	67.7 ± 6.7	64.4 ± 6.3	65.5 ± 8.5	0.600
MIS^*∗*^	7 (4)	5 (5)	7 (4)	6 (3.5)	7 (2.8)	7.5 (4)	0.471
ESA total dose per body weight (U/kg)^*∗*^	3.3 (3.4)	0.3 (0.6)	3.0 (2.4)	4.9 (1.4)	0.5 (1.6)	5.1 (2.1)	**0.001**
Iron per body weight (mg/kg)^*∗*^	15.7 (25)	14.7 (27)	13.4 (16)	16.5 (19)	18.2 (19)	11.7 (20)	0.167
Months of hb < 11 g/dl	6.5 ± 2.3	0.5 ± 0.6	4.3 ± 2.0	9.9 ± 1.7	2.2 ± 2.4	10.4 ± 1.2	**0.001**
10 year survival^*∗*^	21 (77)	21 (56)	21 (77)	53 (76)	21 (69)	53 (77)	0.851

HA: high-amplitude fluctuation; LAH: low-amplitude fluctuation with high hemoglobin; LAL: low-amplitude fluctuation with low hemoglobin; Hb-var (hemoglobin variability); MCV: mean corpuscular volume: RDW: red blood cell distribution width; CRP: C-reactive protein; PTH: parathyroid hormone; URR: urea reduction ratio; MIS: malnutrition-inflammation score; ESA: erythropoiesis-stimulating agents. ^*∗*^Median (Interquartile range); other results are given as mean ± standard deviation.

**Table 3:** Demographic characteristics, biochemical parameters, and medications of patients.

	Exitus (*n*: 31)	Alive (*n*: 138)	*p* value
Age (years)	60.6 ± 20.0	53.4 ± 13.4	**0.027**
Gender (male)	27 (15.9%)	96 (56.8%)	0.248
Duration of hemodialysis (years)	7.6 ± 5.4	9.5 ± 5.7	0.121
Hemoglobin (g/dl)	10.6 ± 1.03	10.8 ± 1.2	0.530
Hb-var (g/dl)^*∗*^	0.94 (0.25)	0.89 (0.26)	0.578
Maximum Hb-var (g/dl)^*∗*^	3.2 (1.7)	3.3 (1.7)	0.565
Coefficient of Hb-var^*∗*^	8.8 (3.1)	8.4 (3.0)	0.429
RDW (%)^*∗*^	16.2 (1.6)	16.3 (2)	0.733
Leukocyte (×10^3^/*µ*l)	7.0 ± 1.7	6.6 ± 1.7	0.354
MCV (fl)	89.7 ± 4.9	90.1 ± 5.5	0.788
Serum Iron (mg/dl)^*∗*^	53.5 (16.3)	58.2 (23.2)	0.206
Transferrin saturation (%)^*∗*^	29.7 (11.7)	29.7 (18.3)	0.301
Ferritin (*µ*g/l)^*∗*^	344.2 (297.7)	305.3 (317.1)	0.478
PTH (pg/ml)^*∗*^	252.1 (419)	415 (699)	0.092
Calcium (mg/dl)	9.1 ± 0.6	9.1 ± 0.7	0.988
Phosphate (mg/dl)	5.1 ± 1.2	5.2 ± 1.3	0.615
Albumin (g/dl)	4.0 ± 0.3	4.2 ± 0.4	0.150
CRP (mg/l)^*∗*^	16.7 (21.0)	8.2 (11.4)	**0.008**
URR (%)	66.5 ± 5.5	66.0 ± 6.6	0.693
MİS^*∗*^	8 (3)	6 (3)	**0.002**
ESA total dose per body weight (U/kg) ^*∗*^	4.1 (2.3)	3.1 (3.8)	0.066
İron per body weight (mg/kg) ^*∗*^	13.2 (23.1)	15.7 (21.5)	0.542
Months of hb < 11 g/dl	6.9 ± 3.1	6.2 ± 3.8	0.379
BMI (kg/m^2^)	21.0 ± 3.8	23.0 ± 4.5	**0.046**
10 year survival^*∗*^	2 (37)	21 (75)	**0.011**

HA: high-amplitude fluctuation; LAH: low-amplitude fluctuation with high hemoglobin; LAL: low-amplitude fluctuation with low hemoglobin; Hb-var (hemoglobin variability); MCV: mean corpuscular volume; RDW: red blood cell distribution width; CRP: C-reactive protein; PTH: parathyroid hormone; URR: urea reduction ratio; MIS: malnutrition-inflammation score; ESA: erythropoiesis-stimulating agents; BMI: body mass index, ^*∗*^Median (interquartile range) other results are given as mean ± standard deviation.
